# Transmission Patterns in a Low HIV-Morbidity State — Wisconsin, 2014–2017

**DOI:** 10.15585/mmwr.mm6806a5

**Published:** 2019-02-15

**Authors:** Katarina M. Grande, Casey L. Schumann, M. Cheryl Bañez Ocfemia, James M. Vergeront, Joel O. Wertheim, Alexandra M. Oster

**Affiliations:** ^1^Division of Public Health, AIDS/HIV Program, Wisconsin Department of Health Services; ^2^Division of HIV/AIDS Prevention, National Center for HIV/AIDS, Viral Hepatitis, STD, and TB Prevention, CDC; ^3^Department of Medicine, University of California, San Diego.

Public health interviews (i.e., partner services), during which persons with diagnosed human immunodeficiency virus (HIV) infection name their sexual or needle-sharing partners (named partners), are used to identify HIV transmission networks to guide and prioritize HIV prevention activities. HIV sequence data, generated from provider-ordered drug resistance testing, can be used to understand characteristics of molecular clusters, a group of sequences for which each sequence is highly similar (linked) to all other sequences, and assess whether named partners are plausible HIV transmission partners. Although molecular data in higher HIV-morbidity states have been analyzed (*1*–*3*), few analyses exist for lower morbidity states (*4*), such as Wisconsin, which reported 4.6 HIV diagnoses per 100,000 persons aged ≥13 years in 2016 (*5*). The Wisconsin Division of Public Health (DPH) analyzed HIV sequence data generated from provider-ordered drug resistance testing and collected through routine HIV surveillance to identify molecular clusters and describe demographic and transmission risk characteristics among pairs of persons whose sequences were highly genetically similar (i.e., molecular linkages). In addition, overlap between partner linkages identified during public health interviews and molecular linkages was assessed. Overall, characteristics of molecular clusters in Wisconsin mirrored those from states with more HIV diagnoses, particularly in that most molecular linkages were observed among persons of the same race (78.2% of non-Hispanic blacks [blacks] linked to other blacks), the same transmission risk (90.2% of men who have sex with men [MSM] linked to other MSM), and the same age group (59.2% of persons aged 20–29 years linked to other persons aged 20–29 years). Among named partner linkages identified during interviews in which both persons also had a reported sequence, overlap of named partner and molecular linkages was moderate: 33.8% of named partners were plausible transmission partners according to available molecular data. Analysis of HIV sequence data is a useful tool for characterizing transmission patterns not immediately apparent using traditional public health interview data, even in a state with lower HIV morbidity. Prevention recommendations generated from national data (e.g., targeting preexposure prophylaxis for HIV-negative persons at high risk and implementing measures to maintain viral suppression among persons with HIV infection) also are relevant in a lower HIV-morbidity state.

HIV sequence data derived from standard drug resistance testing are reportable by laboratories to the Wisconsin DPH and are maintained in a secure surveillance database. HIV-1 sequence data reported in Wisconsin during 2014–2017 for persons with HIV infection diagnosed through August 15, 2017, were analyzed using Secure HIV-TRACE (Secure HIV TRAnsmission Cluster Engine).* This web-based application performed pairwise comparisons of HIV-1 protease and partial reverse transcriptase to measure sequence relatedness and identify sequences that were highly genetically similar at a genetic distance of ≤0.015 substitutions per site (*6*,*7*). Pairs of closely related sequences formed molecular linkages, and a group of ≥2 linked sequences was considered a molecular cluster; these procedures are described elsewhere (*1*,*6*). Weights were applied to persons who had multiple molecular linkages so that each person was counted once (*1*). Analysis also was conducted to describe race/ethnicity, transmission risk, and age at diagnosis among pairs of persons whose sequences were linked (*1*). Multiple imputation using standard surveillance approaches was used to assign a transmission category for persons with missing risk factor information. Findings for linkages by race/ethnicity and transmission category were compared with previously published estimates from national analyses (*1*,*8*).

Named partner data and linkages were obtained through Wisconsin’s PartnerServicesWeb, a CDC-developed database containing the results of public health interviews for persons with diagnosed HIV infection. To compare named partner linkages and molecular linkages, only named partnerships for which both persons had a reported HIV sequence were included in the analysis. These named partner linkages then were matched to the molecular linkages to determine whether named partners also had highly genetically similar sequences. SAS (version 9.3; SAS Institute) was used to conduct all analyses.

Using findings from a national analysis (*1*) as a comparison group, molecular linkages were examined for overall characteristics, sex, race, transmission category, and age partnerships. Among 1,401 persons who had HIV sequences reported to the Wisconsin DPH during 2014–2017, 433 (30.9%) had a molecular linkage to at least one other person (Table 1), representing 703 unique molecular linkages and 119 clusters (range = 2–20 persons per cluster). Among the 433 persons with one or more molecular linkages at the genetic distance threshold of ≤0.015, most were male (88.5%), black (57.3%), MSM (80.8%), and aged 20–29 years (50.3%) (Table 2).

**TABLE 1:** Comparison of human immunodeficiency virus (HIV) molecular clusters* identified in Wisconsin^†^ and HIV molecular clusters — U.S. National HIV Surveillance System (NHSS),^§^ Wisconsin, 2014–2017

Characteristic	No. of molecular clusters identified in Wisconsin	No. of molecular clusters identified in NHSS
No. of persons included in analysis	1,401	40,950
No. of persons with ≥1 molecular linkage	433	12,910
No. of links per person, median (range)	2 (1–13)	1 (1–83)
No. of clusters* in data set (persons per cluster, range)	119 (2–20)	3,584 (2–85)

* A molecular cluster describes a set of ≥2 linked sequences in which each sequence is connected, either directly or indirectly, to all other sequences.

^†^ Analysis included HIV-1 genetic sequences reported through August 15, 2017, to the Wisconsin Division of Public Health for persons with HIV infection diagnosed during 2014–2017.

^§^ https://www.ncbi.nlm.nih.gov/pubmed/26302431.

**TABLE 2:** Comparison of persons identified as part of human immunodeficiency virus (HIV) molecular clusters* in Wisconsin^†^ and persons identified as part of HIV molecular clusters — U.S. National HIV Surveillance System (NHSS),^§^ Wisconsin, 2014–2017

Characteristic	No. of persons identified as part of molecular clusters in Wisconsin (%)	No. of persons identified as part of molecular clusters in NHSS (%)
**Total persons with ≥1 molecular linkage**	**433 (100)**	**12,910 (100)**
**Sex**		
Male	383 (88.5)	11,232 (87.0)
Female	50 (11.5)	1,678 (13.0)
**Race/Ethnicity^¶^**		
Black, non-Hispanic	248 (57.3)	5,445 (42.2)
White, non-Hispanic	112 (25.9)	3,992 (30.9)
Hispanic/Latino^¶^	55 (12.7)	2,884 (22.3)
Other**	18 (4.2)	589 (4.6)
**Transmission category^††^**		
MSM	350 (80.8)	9,839 (76.2)
MSM who inject drugs	15 (3.5)	496 (3.8)
Men who inject drugs	5 (1.2)	309 (2.4)
Women who inject drugs	9 (2.1)	268 (2.1)
Heterosexual males	11 (2.5)	583 (4.5)
Heterosexual females	39 (9.0)	1,409 (10.9)
Other	3 (0.7)	6 (0.5)
**Age at HIV diagnosis (yrs)**		
<13	3 (0.7)	N/A
13–19	53 (12.2)	1,162 (9.0)
20–29	218 (50.3)	5,954 (46.1)
30–39	74 (17.1)	3,172 (24.6)
40–49	53 (12.2)	1,841 (14.3)
50–59	29 (6.7)	656 (5.1)
≥60	3 (0.7)	125 (1.0)

**Abbreviations:** MSM = men who have sex with men; N/A = not applicable.

* A molecular cluster describes a set of ≥2 linked sequences in which each sequence is connected, either directly or indirectly, to all other sequences.

^†^ Analysis included HIV-1 genetic sequences reported through August 15, 2017, to the Wisconsin Division of Public Health for persons with HIV infection diagnosed during 2014–2017.

^§^ https://www.ncbi.nlm.nih.gov/pubmed/26302431.

^¶^ Hispanics/Latinos can be of any race.

** Persons of other races/ethnicities include Asian, American Indian/Alaska Native, and multiple races.

^††^ Data have been statistically adjusted to account for missing transmission category using multiple imputation; therefore, values might not sum to column totals.

Analysis of molecular partnerships by race/ethnicity revealed that blacks and non-Hispanic whites most commonly linked with persons of their own racial group (78.2% and 54.5%, respectively), whereas a minority of Hispanic/Latino persons linked with other Hispanics/Latinos (31.7%) (Table 3). Partnerships by transmission category indicated that MSM most commonly had molecular linkages with other MSM (90.2%) (Table 3). MSM who injected drugs also were primarily linked to MSM (88.3%).

**TABLE 3:** Comparison of potential transmission partnerships identified in human immunodeficiency virus (HIV) molecular clusters* in Wisconsin^†^ and potential transmission partnerships — U.S. National HIV Surveillance System (NHSS),^§^ Wisconsin, 2014–2017

Characteristic	Molecular clusters identified in Wisconsin		Molecular clusters identified in NHSS	
	Total no. of persons	No. of partnerships (row %)	Total no. of persons	No. of partnerships (row %)
**Same-race partnerships^¶^**				
Black, non-Hispanic	248	194 (78.2)	5,445	4,410 (81.0)
White, non-Hispanic	112	61 (54.5)	3,992	2,475 (62.0)
Hispanic/Latino**	55	17 (31.7)	2,884	1,500 (52.0)
**Transmission category^††^ partnerships**				
Among MSM, linkages to MSM	350	316 (90.2)	9,839	8,658 (88.0)
Among MSM who inject drugs, linkages to MSM	15	13 (88.3)	496	377 (76.0)
Among heterosexual females, linkages to MSM	39	12 (31.5)	1,409	409 (29.0)
**Same-age group^§§^ partnerships (yrs)**				
<13	3	0 (0.0)	N/A	N/A
13–19	53	8 (15.1)	N/A	N/A
20–29	218	129 (59.2)	N/A	N/A
30–39	74	12 (16.2)	N/A	N/A
40–49	53	8 (15.1)	N/A	N/A
50–59	29	6 (20.7)	N/A	N/A
≥60	3	0 (0.0)	N/A	N/A
**Same-age group^§§^ partnerships of black MSM (yrs)**				
<13	0	0 (0.0)	N/A	N/A
13–19	42	8 (19.0)	N/A	N/A
20–29	123	71 (57.7)	N/A	N/A
30–39	30	6 (20.0)	N/A	N/A
40–49	10	1 (10.0)	N/A	N/A
50–59	4	0 (0.0)	N/A	N/A
≥60	0	0 (0.0)	N/A	N/A

**Abbreviations:** MSM = men who have sex with men; N/A = not applicable.

* A molecular cluster describes a set of ≥2 linked sequences in which each sequence is connected, either directly or indirectly, to all other sequences.

^†^ Analysis included HIV-1 genetic sequences reported through August 15, 2017, to the Wisconsin Division of Public Health for persons with HIV infection diagnosed during 2014–2017.

^§^ https://www.ncbi.nlm.nih.gov/pubmed/26302431.

^¶^ Persons of other races represented <5% of the clustered sample and were not analyzed independently.

** Hispanics/Latinos can be of any race.

^††^ Data have been statistically adjusted to account for missing transmission category using multiple imputation; therefore, values might not sum to column totals.

^§§^ Age group is based on the person’s age at HIV diagnosis. Same-age group partnerships were not assessed in Oster et al. https://www.ncbi.nlm.nih.gov/pubmed/26302431.

Persons aged 20–29 years at diagnosis, the largest age group in the data set, were most likely to have molecular linkages with others aged 20–29 years (59.2%) (Table 3). Persons aged 13–19 years also were commonly linked with persons aged 20–29 years (58.2%). Among the 123 black MSM aged 20–29 years, 57.7% were molecularly linked to other persons aged 20–29 years (Table 3), and 19.2% were linked to persons aged 13–19 years. Among 139 named partner linkages identified during public health interviews in which both persons each had a reported sequence, 47 (33.8%) also had a molecular linkage, indicating that the named partners were plausible transmission partners.

## Discussion

These findings from Wisconsin, that approximately one of every three persons with a reported HIV sequence was molecularly linked to at least one other person, largely align with those found in a national analysis, for which most data originated from states with higher HIV morbidity (*1*,*8*). The Wisconsin data also revealed that most molecular linkages occurred among persons of the same racial/ethnic, transmission risk, and age groups, with the highest percentages of same partnerships observed among blacks, MSM, and persons aged 20–29 years. These findings also were consistent with findings from the national analysis (*1*,*8*) and validate the generalizability of characteristics of national molecular surveillance data to Wisconsin. Therefore, surveillance strategies to combine sequence data and interview data in identifying clusters are equally useful in states with lower HIV morbidity.

It is important to note that directionality cannot be inferred from molecular surveillance data alone, nor is it the intent of molecular cluster analysis to confirm transmission relationships. Rather, the patterns of persons with genetically related sequences are helpful in viewing population-level patterns of transmission and guiding prevention activities.

The findings in this report are subject to at least four limitations. First, the molecular clusters identified do not include all persons in the transmission network because not all persons with HIV infection know their status, some with diagnosed infection are not linked to HIV medical care, and some linked to care did not receive antiretroviral resistance testing or did not have their sequence reported. Second, in states with long-standing molecular reporting, two thirds of persons who are linked to care within 3 months of diagnosis have received drug resistance testing, although this linkage is less likely among older persons and black persons, and in areas with smaller populations (*9*). The demographics of persons who did not receive resistance testing were not assessed in the Wisconsin data set but could be a limitation if the national linkage biases exist in Wisconsin. Third, the comparison of molecular linkages with named partner linkages was limited to persons who named partners and might not be representative of all persons with HIV infection in Wisconsin. Finally, imputation was used for persons with missing risk information (13%), which could affect the estimates.

Because most new diagnoses of HIV infection in Wisconsin occur in clinical outpatient settings rather than testing sites (*10*), it is common for a person with newly diagnosed HIV infection to already be established in care and have had resistance testing completed by the time a public health interview is conducted. This situation makes it possible for public health personnel to prioritize follow-up and intensive prevention measures (e.g., referral and linkage to preexposure prophylaxis for HIV-negative partners at high risk) for members of rapidly expanding clusters and their partners. Despite relatively low overlap between molecular data and named partner data, the results of public health interviews are still important for identifying persons at high risk for acquiring HIV infection, identifying undiagnosed HIV infection, and ensuring that persons with diagnosed HIV infection are engaged in HIV medical care. The combination of public health interview and molecular sequence data is a powerful new tool for understanding HIV transmission networks and identifying population- or individual-level interventions to reduce HIV transmission and improve health outcomes.

**Summary**

**What is already known about this topic?**

Identifying named partners through public health interviews is an important strategy for interrupting human immunodeficiency virus (HIV) transmission. Analyzing HIV molecular sequence data also can identify networks of potential transmission partners.

**What is added by this report?**

Most molecular linkages in Wisconsin were among persons within the same racial/ethnic, risk, and age groups. Among named partner linkages where both persons had an HIV sequence available, 33.8% also had a molecular linkage and were deemed plausible transmission partners.

**What are the implications for public health practice?**

Supplementing named partner data with molecular data might detect HIV transmission networks not elucidated through traditional public health interviews and identify opportunities for prevention in rapidly growing clusters of HIV infections in states with lower HIV morbidity.

## Footnotes

* Secure HIV-TRACE is a web-based tool developed by CDC with the University of California, San Diego and Temple University to detect, analyze, and visualize HIV molecular clusters.
